# Use of TD-GC–TOF-MS to assess volatile composition during post-harvest storage in seven accessions of rocket salad (*Eruca sativa*)

**DOI:** 10.1016/j.foodchem.2015.08.043

**Published:** 2016-03-01

**Authors:** Luke Bell, Natasha D. Spadafora, Carsten T. Müller, Carol Wagstaff, Hilary J. Rogers

**Affiliations:** a Department of Food and Nutritional Sciences, University of Reading, Whiteknights, PO Box 226, Reading, Berkshire RG6 6AP, UK; b Cardiff School of Biosciences, Cardiff University, Main Building, PO Box 915, Cardiff, CF10 3TL, UK; c Centre for Food Security, University of Reading, Whiteknights, Reading, Berkshire RG6 6AP, UK

**Keywords:** *Eruca sativa*, *Diplotaxis tenuifolia*, *Brassicaceae*, Rocket salad, Rucola, Gas chromatography mass spectrometry, Volatile organic chemicals, Isothiocyanates

## Abstract

An important step in breeding for nutritionally enhanced varieties is determining the effects of the post-harvest supply chain on phytochemicals and the changes in VOCs produced over time. TD-GC–TOF-MS was used and a technique for the extraction of VOCs from the headspace using portable tubes is described. Forty-two compounds were detected; 39 were identified by comparison to NIST libraries. Thirty-five compounds had not been previously reported in *Eruca sativa.* Seven accessions were assessed for changes in headspace VOCs over 7 days. Relative amounts of VOCs across 3 time points were significantly different – isothiocyanate-containing molecules being abundant on ‘Day 0’. Each accession showed differences in proportions/types of volatiles produced on each day. PCA revealed a separation of VOC profiles according to the day of sampling. Changes in VOC profiles over time could provide a tool for assessment of shelf life.

## Highlights

• We present a robust method for VOC analysis from rocket salad packaging headspace.
• TD-GC–TOF-MS putatively identified 39 volatile compounds.
• VOC profiles for *Eruca sativa* varied significantly between accessions.
• Isothiocyanate compounds degrade significantly over shelf-life.
• Changes in VOC profiles could provide a useful tool for assessment of leaf quality.

## 1 Introduction

Rocket (or arugula, rucola, roquette) species are of increasing commercial importance across the world. Leaves of the crop are usually sold in mixed salad bags or whole bags, and in some niche markets as gourmet microleaves. The nutritional and sensory quality of leaves throughout the supply chain is of major concern to producers and supermarkets, as they will ultimately be accepted or rejected, by the consumer, based on these attributes. Much of the current rocket supply chain is designed to preserve predominantly visual and morphological traits of leaves (such as stem browning/yellowing) before they reach the consumer. Very little research has been conducted to determine the phytochemical and volatile organic compound (VOC) losses incurred post-harvest (Verkerk, Dekker, & Jongen, 2001).

Rocket varieties are genetically very diverse, and morphological uniformity can be an issue for plant breeders and growers alike. One plant can have very different leaf shapes from another, even within the same variety (Egea-Gilabert et al., 2009). Rocket species are generally preferential outbreeders, making production of uniform breeding lines difficult. This variability has been shown to extend to concentrations of phytochemicals (Bell, Oruna-Concha, & Wagstaff, 2015), where significant differences in glucosinolate (GSL) and flavonols have been detected amongst accessions. An important step in breeding for nutritionally enhanced varieties is determining the effects of the post-harvest supply chain on phytochemicals and the changes in volatile degradation products produced over time. The concentrations and/or relative abundances of both of these, which include isothiocyanates, (ITCs) may also vary greatly, depending on the levels of physical damage during processing of the leaves. The VOC bouquet is the term used to describe the collection of volatiles within the headspace of a plant or other foodstuff, often giving rise to aromas. These aromas will affect the sensory attributes perceived by the consumer when the product is eaten, and they influence re-purchase (Ragaert, Verbeke, Devlieghere, & Debevere, 2004). This may have consequences on consumers’ nutritional intake, and hence long-term health.

VOCs found in rocket comprise ITCs, alkanes, aliphatic alcohols, carbonyl compounds, fatty acids, esters, phenols and C_13_-norisoprenoids (Blazevic & Mastelic, 2008). However, comparison of the relative abundance amongst cultivars has not been established, as earlier studies only utilized one commercially bought, bagged variety, and leaves from a wild population (Blazevic & Mastelic, 2008; Jirovetz, Smith, & Buchbauer, 2002). Given the very different sample sources, differences between the two studies may be representative of environmental stresses, as well as genetic variation (Varming et al., 2004). These include exposure to fungal diseases, wounding (pre- and post-harvest), and variations in temperature and humidity during growth and while in controlled environment conditions – all of which can lead to changes in phytochemical content and VOCs produced (Schouten et al., 2009).

In this study rocket salad was grown under controlled environment conditions, thus reducing environmental stress responses, enabling effects of post-harvest storage on VOC profiles to be assessed. Thermal desorption gas chromatography time-of-flight mass spectrometry (TD-GC–TOF-MS) was used to determine changes in VOCs during storage of seven different accessions, demonstrating that collection of headspace volatiles onto thermal desorption tubes is a rapid and robust method for assessing post-harvest changes and identifying differences in these changes amongst accessions.

## 2 Materials and methods

### 2.1 Plant material

Six *Eruca sativa* accessions were obtained from European gene banks, four from the Leibniz-Institut für Pflanzengenetik und Kulturpflanzenforschung (IPK Gatersleben, Germany), one from the Centre for Genetic Resources in the Netherlands (CGN, Wageningen, The Netherlands) and one from The University of Warwick Crop Centre Genetic Resources Unit (Wellesbourne, UK; formerly Warwick HRI). The Elsoms Seeds variety SR3 was used as a commercial comparator. Accessions have been coded to protect commercially sensitive information.

### 2.2 Growing conditions and simulated shelf life

Each accession was germinated under controlled environmental conditions (Fitotron, Weiss-Technik UK, Loughborough, UK). Long-day lighting was used (16 h light, 8 h dark) at an intensity of 200 μmol m^−2^ s^−1^. Day temperatures were set at 20 °C and night temperatures at 14 °C. Seedlings were grown for 10 days in seedling trays and then transplanted to larger trays. Subsequently, plants were grown for a further 20 days and then leaves were harvested at 30 days. Leaves were collected in batches of 70 g in three experimental replicates per accession. Amount of leaf material was chosen based on preliminary experiments, where abundant yields of VOCs were obtained.

### 2.3 Sample collection

Whole leaves were placed into a multi-purpose roasting bag (25 cm × 38 cm, TJM Ltd.) and sealed, using an elastic band and an Eppendorf tube with the end cut off, which served as a sampling port for the TD tubes (see supplementary material for diagram). Leaves were then disrupted manually within the bags for 10 s by crushing the leaves between the hands and making a vigorous rubbing motion. Care was taken not to perforate the bags and inadvertently release VOCs. Three replicates were taken for each sample, including three ‘blank’ samples of atmosphere within empty bags to rule out any possible contaminating VOCs. These were prepared and sampled in an identical fashion, with the exception that no leaves were contained in the bag.

### 2.4 Post-harvest storage simulation

Harvested rocket leaves were stored in a dark, controlled temperature room at 4 °C, to simulate industrial storage conditions for 7 days. Bags were only removed from the environment while samples were taken (room temperature, ∼22 °C).

### 2.5 TD-GC–MS-TOF analysis

All tubes were desorbed by a TD100 thermal desorption system (Markes International Ltd., Llantrisant, Wales, UK), using the following settings for tube desorption: 5 min at 100 °C, followed by 5 min at 280 °C, trap flow of 40 ml/min and trap desorption and transfer: 20 °C/s to 300 °C, split flow of 20 ml/min into GC (7890A; Agilent Technologies, Inc., Stockport, UK). VOCs were separated over 60 min, 0.32 mm ID, 0.5 μm film thickness Rxi-5ms (Restek) at 2 ml continuous flow of helium, using the following temperature programme: initial temperature 40 °C for 2 min, 5 °C/min to 240 °C, final hold 5 min. The BenchTOF-dx mass spectrometer (Almsco International, Cincinnati, OH, USA) was operated in EI mode at an ion source temperature of 275 °C and a mass range of 35–350 *m*/*z*. A retention time standard (C8–C20, Sigma Aldrich, Gillingham, UK) was prepared by injection of 1 μl of the standard mixture directly onto a thermal desorption tube, and analysed under the same conditions as the samples.

Data from GC–MS measurements were processed with MSD ChemStation software (E.02.01.1177; Agilent Technologies, Inc., Stockport, UK) and deconvoluted and integrated with AMDIS (NIST 2011), using a custom retention-indexed mass spectral library. MS spectra from deconvolution were searched against the NIST 2011 library (Mallard, Sparkman, & Sparkman, 2008) and only compounds scoring >80% in forward and backward fit were included into the custom library. Putative identifications were based on match of mass spectra (>80%) and retention index (RI ± 15) (Beaulieu & Grimm, 2001).

Compounds abundant in controls or in only one of the three replicates were excluded from statistical analyses. Areas of remaining compounds were normalized to total area of chromatograms prior to averaging within samples.

### 2.6 Statistical analysis

Results from the three biological replicates for each time point were averaged and analysed using XL Stat software (Addinsoft, New York City, New York, USA). ANOVA + Tukey’s HSD test (*p* ⩽ 0.05) and principal component analyses (PCA; Pearson *n* − 1) were performed on the data to determine significant differences and correlations of compounds detected over time and on each respective sampling day. Tukey’s test was chosen because of its high stringency (compared to Fisher’s test, for example) and a reduced possibility of Type I statistical errors.

## 3 Results and discussion

### 3.1 Composition and functional analysis of *E. sativa* VOC bouquet

We focussed on *E. sativa* accessions to assess the range of VOCs produced by different genotypes from the same species, and then to determine whether, within this narrower genetic range, there were sufficient VOC differences to discriminate amongst accessions. A total of 39 compounds were putatively identified by comparison to NIST libraries, and a further three unknown volatile compounds were also detected (see Table 1 for compounds, their identification codes, retention indices and CAS numbers). Compounds comprised several classes of aliphatic organic compounds, including alcohols, aldehydes, ketones, isothiocyanates and furanones; some of which have been reported in other studies (Blazevic & Mastelic, 2008; Jirovetz et al., 2002). However, to our knowledge, only four of these compounds have been previously reported in *E. sativa* (**C8**, **C14**, **C20** and **C38**). Fifteen of the compounds have been detected in other members of the *Brassicaceae* family. These include broccoli (*Brassica oleracea* var. *italica*) (**C10**, **C15**, **C9**) (Hansen, Laustsen, Olsen, Poll, & Sorensen, 1997), radish (*Raphanus sativus*) (**C8**, **C20**, **C33**, **C25**) (Blazevic & Mastelic, 2009), kale (*B. oleracea* var. *acephala*) (**C3**, **C10**, **C8**, **C31**, **C20**, **C29**) (Fernandes, de Pinho, Valentao, Pereira, & Andrade, 2009), oilseed rape (*Brassica napus*) (**C20**, **C6**, **C7**), Thale cress (*Arabidopsis thaliana*) (**C20**, **C29**, **C3**, **C9**, **C14**) (Rohloff & Bones, 2005) and mustard (*Brassica juncea*) (**C15**, **C9**, **C14**, **C23**) (Zhao, Tang, & Ding, 2007).

### 3.2 Relative VOC abundance and differences amongst rocket gene bank accessions over simulated shelf life

#### 3.2.1 Across-day variation

**Fig. 1:**
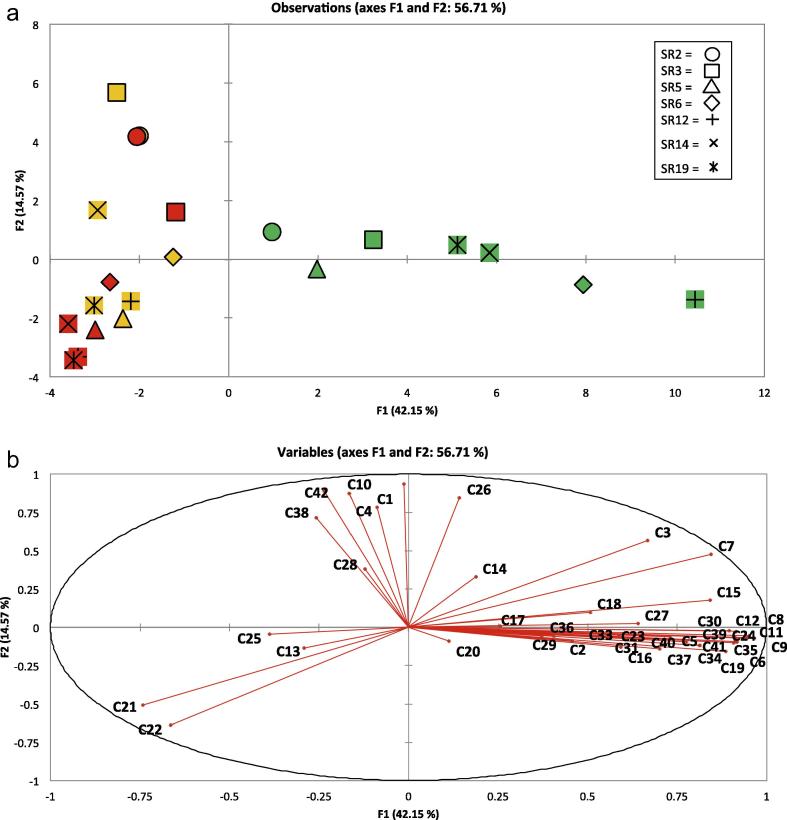
Loadings plot (a) and scores plot (b) from principal components analysis of seven accessions of *E. sativa* and the volatile organic compounds identified. Data points were averaged for each accession time point (consisting of three replicates). PC1 vs. PC2 (F1 and F2) accounted for 56.72% of the total variation within data. For compound identities refer to Table 1. SR codes refer to each of the 7 accessions used in the experiment (SR2, SR3, SR5, SR6, SR12 SR14 and SR19) with the following colour coding, 0 (green), 3 (orange) and 7 (red) corresponding to the day of sampling.

The abundance of the 42 VOCs was determined in each of the seven *E. sativa* accessions (Table 2) over three different storage time-points: ‘Day 0’ (at harvest) and following +3 and +7 days of storage at a commercially relevant temperature of 4 °C. Seven days is the typical time taken from harvest to retail (Favell, 1998), assuming a typical supply chain where rocket is imported by lorry from northern or southern Italy (depending on the season) to the UK. Thirty-one of the 42 compounds detected were significantly different in percentage-abundance amongst rocket accessions, across the three sampling days (ANOVA with *post hoc* Tukey’s HSD test; see Table 2). Fig. 1 (PCA loadings plot) shows how each of the genotypes is arranged spatially, according to the volatiles produced on each sampling day. On ‘Day 0’ there is a large degree of separation along the F1 axis, indicating that genotypic variability is high in terms of the types of volatiles produced and their relative abundance. Samples SR2 and SR12 are the most dissimilar in this respect with varying degrees of similarity with the other five genotypes. This indicates that there may be a high degree of genetic variability and control involved in VOC production in the early stages of shelf life, as environmental variation was minimal during plant growth and sampling. On ‘Day 3’ and ‘Day 7’, the distinction between accessions is somewhat reduced, and volatile profiles less variable for each respective accession. The profiles of SR2 on ‘Day 3’ and ‘Day 7’ are virtually indistinguishable in Fig. 1a, and similarly with SR5.

#### 3.2.2 Within-day variation

##### 3.2.2.1 General

A separate ANOVA Tukey’s HSD test (*p* ⩽ 0.05) was performed on data from each of the three sampling days. Several significant differences were observed between cultivars on each day. Twelve compounds were significantly different between accessions on ‘Day 0’, nine on ‘Day 3’ and six on ‘Day 7’. The dwindling abundances of VOCs in the latter shelf-life samples would seem to indicate the depletion of GSLs and other defense related compounds. The most interesting of these are discussed in this section.

##### 3.2.2.2 Isothiocyanates

On ‘Day 0’ SR6 and SR5 were the only accessions where 4-isothiocyanato-1-butene (**C2**, both 0.1 ± <0.1%) was detected. 4-methylpentyl ITC (**C20**) was detected in all accessions with SR5 being significantly different (4.3 ± 1.0%) from all others with the exception of SR19 (2.4 ± 0.5%). SR5 contained over six times greater abundance of this ITC than SR3.

Hexyl ITC (**C29**) was similarly detected in all accessions (‘Day 0’), with SR5 having the highest abundance (1.8 ± 0.7%) – significantly different in this case from SR19, which had the lowest recorded abundance (0.2 ± 0.1%; nine times less abundant in relative terms). Bell et al. (2015) found that SR5 had high total GSL concentrations (11.5 mg g^−1^ DW) compared with other *Eruca* and *Diplotaxis* accessions, potentially making it a valuable source of genetic material for breeding programmes interested in enhancing GSL/ITC accumulation traits.

##### 3.2.2.3 Alcohols

(Z)-2-penten-1-ol (**C9**) was detected in all accessions except SR5, with the highest abundance detected in SR12 (0.3 ± 0.1%; ‘Day 0’) which was significantly different from zero (i.e. significantly different from samples with 0% abundance). Other alcohols, such as 1-penten-3-ol (**C3**) displayed no significant differences on this sampling day. Some of these differences may indicate a genetic component to VOC production, i.e. the types and abundances produced may be under direct genetic regulation. Alcohols are typically used by plants as a defensive mechanism (Ruther & Kleier, 2005), and often provide the ‘cut grass’ aroma found in leafy vegetables. Plant defence mechanisms are known to rely on genetic regulation via enzymatic regulation in other species (D’Auria, Pichersky, Schaub, Hansel, & Gershenzon, 2007), and it is reasonable to assume that the same may be true for rocket. D’Auria et al. (2007), showed this to be the case in *Arabidopsis* for production of (Z)-3-hexen-1-yl acetate via a BAHD acetyltransferase enzyme. It is also widely known that nitriles, epithionitriles and thiocyanates are produced enzymatically in *Brassicaceae* through specifier proteins in the GSL-myrosinase reaction (Kuchernig, Backenköhler, Lübbecke, Burow, & Wittstock, 2011).

##### 3.2.2.4 Sulphur aromatic compounds

3-Ethyl-thiophene (**C37**) was detected in only two accessions, SR6 and SR12 (both <0.1 ± <0.1%, ‘Day 0’) and were significantly different from each other, despite the exceedingly small abundances observed. Tetrahydrothiophene (**C38**) was detected in every accession, but SR5 had the highest abundance (1.2 ± 0.1%, ‘Day 0’) which was significantly different from the other samples. It is likely that these types of VOCs also contribute to the characteristic pungent/mustard/pepper sensory attributes of rocket, as well as ITCs.

By ‘Day 7’, pyrrolidine-1-dithiocarboxylic acid 2-oxocyclopentyl ester (**C36**) was detected in only two accessions: SR5 (0.4 ± 0.2%) and SR6 (2.9 ± 1.3%). Tetrahydrothiophene (**C38**) was present in all accessions with the exception of SR12 and SR14.

##### 3.2.2.5 Imines

On ‘Day 3’ 5-nonanone-oxime (**C21**) was most abundant in SR5 (36.9 ± 7.5%), significantly higher than in either SR3 or SR6 (8.7 ± 2.8%; 11.1 ± 2.8%). Abundances were generally much higher across all accessions than on ‘Day 0’, though with fewer significant differences between accessions, possibly because of more variation between replicates. SR3 also contained the lowest abundance of *O*-methyloxime-butanal (**C22**) (4.2 ± 1.8%). In contrast, SR19 had the highest abundance (30.9 ± 0.6%) of this compound.

##### 3.2.2.6 Aldehydes

A very large range of abundances was observed for (E)-4-oxohex-2-enal (**C1**) amongst accessions. This compound was not observed at all in SR5 on ‘Day 3’, yet accounted for 49.9 ± 0.9% of VOCs produced by SR3 on this sampling day. SR3 also contained significantly higher abundance of (E)-2-pentenal (**C10**) (4.0 ± 0.8%) than did SR5 (0.8 ± 0.6%) on ‘Day 3’. In contrast, a narrow range of abundance was observed for 2-hexenal (**C7**); SR19 had the lowest (1.0 ± 0.5%) and SR2 the highest (6.5 ± 1.6%), with both being the only accessions significantly different from each other.

The more exotic aldehyde (E,E)-2,4-hexadienal (**C11**) was only observed in SR12 (0.3 ± 0.1%) on ‘Day 3’. These observations are interesting, in contrast with ‘Day 0’, where no significant differences were observed in any of the aldehyde compounds that were present. Despite the apparent differences in the percentage abundances in compound **C1** (ranging from 2.5% to 22.4%), the fact that no significant differences were observed can most likely be explained by the very large standard errors of each accession, and the highly variable nature of VOC production. It might suggest, however, that some varieties are more genetically predisposed to differing rates of lipid oxidation, that are characteristic of plants kept in storage (Varming et al., 2004).

**Fig. 2:**
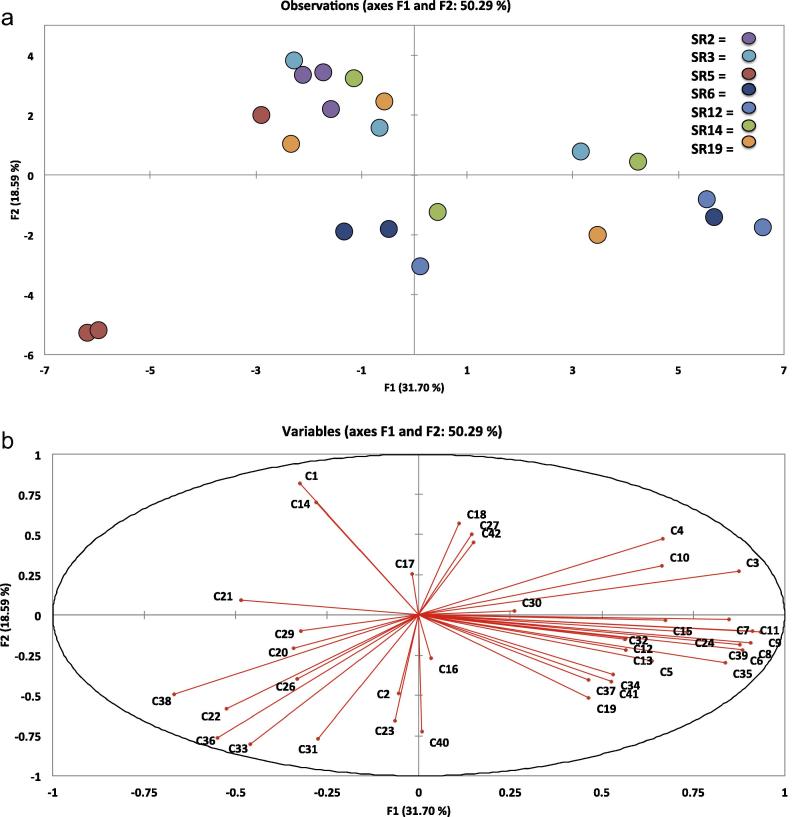
Loadings plot (a) and scores plot (b) from principal components analysis of seven accessions of *E. sativa*, and the volatile organic compounds identified on ‘Day 0’. Individual replicates were plotted to visualize variation within and between accessions. PC1 vs. PC2 (F1 and F2) accounted for 50.29% of the total variation within data. For compound identities refer to Table 1. SR codes refer to each of the seven accessions used in the experiment (SR2, SR3, SR5, SR6, SR12, SR14 and SR19). See inset for accession colour coding.

**Fig. 3:**
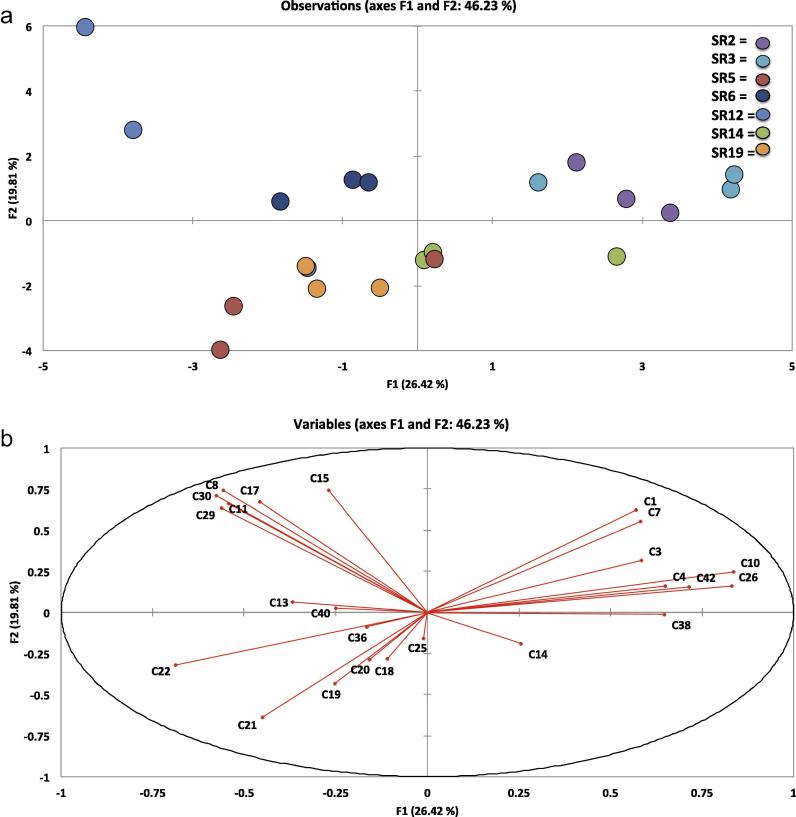
Loadings plot (a) and scores plot (b) from principal components analysis of seven accessions of *E. sativa*, and the volatile organic compounds identified on ‘Day 3’. Individual replicates were plotted to visualize variation within and between accessions. PC1 vs. PC2 (F1 and F2) accounted for 46.23% of the total variation within data. For compound identities refer to Table 1. SR codes refer to each of the seven accessions used in the experiment (SR2, SR3, SR5, SR6, SR12, SR14 and SR19). See inset for accession colour coding.

**Fig. 4:**
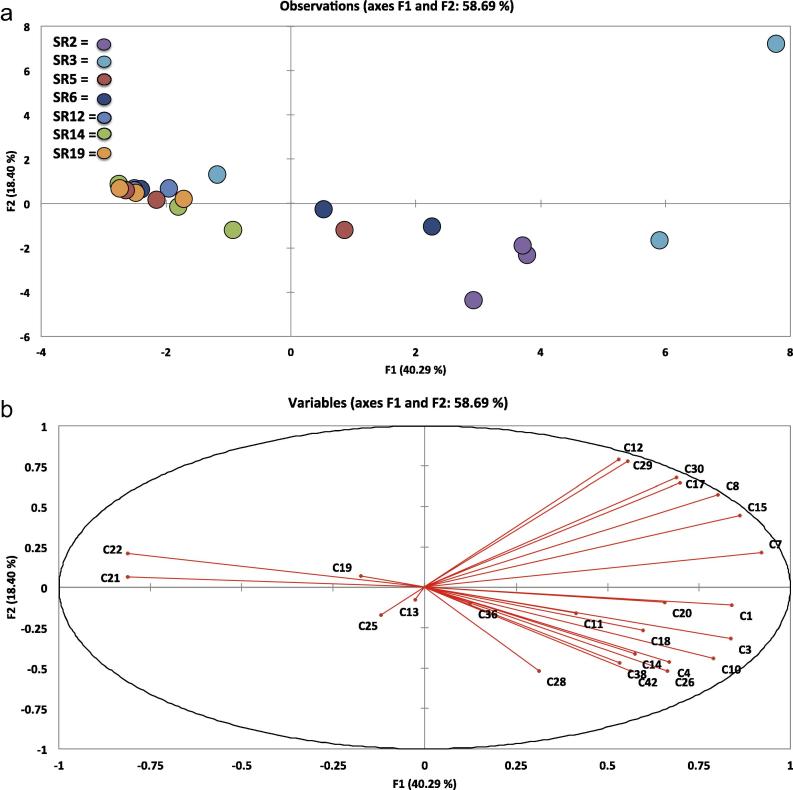
Loadings plot (a) and scores plot (b) from principal components analysis of seven accessions of *E. sativa*, and the volatile organic compounds identified on ‘Day 7’. Individual replicates were plotted to visualize variation within and between accessions. PC1 vs. PC2 (F1 and F2) accounted for 58.69% of the total variation within data. For compound identities refer to Table 1. SR codes refer to each of the seven accessions used in the experiment (SR2, SR3, SR5, SR6, SR12, SR14 and SR19). See inset for accession colour coding.

Figs. 1–4, which display the PCA plots, also show how individual replicates from each accession vary in VOC abundance and profile within each sampling day. In some cases this can be quite marked, illustrating how profiles can be affected by multiple and very small factors, despite efforts to maintain constant experimental conditions. Further independent experiments will allow better elucidation and confirmation of VOC profiles and relative abundances in rocket in terms of within-day and within-cultivar production.

### 3.3 Correlation of VOC abundance with shelf-life time points: PCA

#### 3.3.1 General

PCA of VOCs across and within the three time-points revealed significant correlations between accessions and the prevalence of different types of compounds during storage, indicating sizeable variation amongst the seven accessions. All data are represented as sample scores and loadings plots (Figs. 1–4).

#### 3.3.2 Across-day PCA

PC1 vs. PC2 (F1 and F2; Fig. 1) accounted for 56.72% of the total variation within data and *r*-values became significant at ±0.434 (*p* ⩽ 0.05). ‘Day 0’ samples formed a distinct, linear cluster (green; Fig. 1a) spanning the first principal component. In contrast, however, ‘Day 3’ (yellow) and ‘Day 7’ (red) samples were not separable and both formed a linear cluster along the second principal component (F2).

Table 2 illustrates where significant differences between compounds for each accession were observed. When compared with the scores plot, it can be seen that a distinct cluster of volatiles is highly correlated with ‘Day 0’ along the first principal component, and two other separate localizations of VOCs can be seen correlating in the top and bottom left of Fig. 1b. This suggests that there may be some genotypic differentiation, in terms of the volatiles produced on both ‘Day 3’ and ‘Day 7’. Compounds with strong correlations along the first principal component include (Z)-2-penten-1-ol (**C9** *r* = 0.950), (E)-2-hexenal (**C8** *r* = 0.946), (E,E)-2,4-hexadienal (**C11** *r* = 0.964), 2-methyl-2-butanal (**C6** *r* = 0.916), 5-ethyl-2(5H)-furanone (**C12** *r* = 0.915), 4-methyl-2-(2-methyl-1-propenyl)-pyridine (**C35** *r* = 0.907), ethylidene-cyclopropane (**C24** *r* = 0.912) and 5-methyl-4-hexen-3-one (**C19** *r* = 0.886).

Compounds correlating with accessions along the second principal component, and with SR3 and SR2, on ‘Day 3’ and ‘Day 7’ consist of vinylfuran (**C42** *r* = 0.874), (E)-4-oxohex-2-enal (**C1** *r* = 0.889) and tetrahydrothiophene (**C38** *r* = 0.716).

The presence of aldehydes within the ‘Day 3’ and ‘Day 7’ clusters is consistent with extensive and prolonged lipid degradation over the shelf life period. The exact role of many of these VOCs is unknown, and the exact significance they may have in affecting human sensory attributes when rocket is consumed is similarly not well understood.

#### 3.3.3 Within-day PCA

##### 3.3.3.1 ‘Day 0’

The first two principal components (F1 and F2; Fig. 2) explained 50.29% of the total variation present within the sample set and correlations between accessions became significant at *r* = ±0.433 (*p* ⩽ 0.05). Four clusters are apparent in the loadings plot (Fig. 2a); however, only one of these clusters contains all three respective sample replicates for the accessions (SR2). This spread of individual replicates across clusters indicates that VOC production for different germplasm accessions is inherently variable, even within genotypes.

The cluster located in the top left of the plot contains all three replicates of SR2, indicating a high degree of uniformity in terms of VOC production on the initial sampling day. This cluster also contains two replicates of accessions SR3 and SR19. It is interesting to note that the commercial accession (SR3) displays more variation than do many of the ‘wild’ germplasm accessions. It would be expected that commercial cultivars would be more uniform than cultivars of open pollinated accessions, as they (in theory) would have had at least some rudimentary plant breeding before commercial sale. Artificial selections would have been made in a breeding programme to select plants that had desirable characteristics, such as a pungent odour. Throughout successive generations, one would expect the variation associated with such a trait to decrease, but that does not appear to be the case and is perhaps indicative of little concerted breeding to improve varietal uniformity. When compared with Fig. 2b, it can be seen that two compounds in particular are correlated with this cluster: (E)-4-oxohex-2-enal (**C1** *r* = 0.816) and 3-hexen-1-ol (**C14** *r* = 0.699). The former of these was highlighted in the previous section as being an abundant compound in accessions, but on sampling ‘Day 3’ and ‘Day 7’.

The second cluster to the extreme right of Fig. 2a is much less compact, containing two replicates of SR12 and individual replicates of SR3, SR14, SR6 and SR19. Compounds strongly correlated in this position along the principal component in Fig. 2b are much more numerous and diverse, including (Z)-2-penten-1-ol (**C9** *r* = 0.934), (E,E)-2,4-hexadienal (**C11** *r* = 0.911), (E)-2-hexenal (**C8** *r* = 0.907), 2-methyl-2-butanal (**C6** *r* = 0.884), 4-methyl-2-(2-methyl-1-propenyl)-pyridine (**C35** *r* = 0.837), 2-hexenal (**C7** *r* = 0.847) and ethylidene-cyclopropane (**C24** *r* = 0.733). These compounds are indicative of ‘Day 0’ VOCs and seem to be produced in most abundance at the initial point of tissue damage, with levels declining or completely disappearing on subsequent sampling days.

The third cluster (located centrally and almost equidistant from clusters one and two) contains two replicates of SR6 and single replicates of SR14 and SR12. Compounds correlated in this vicinity of the scores plot are much more loosely distributed and have no strong correlation with either principal component. The presence of ITC compounds towards the lower section of the analysis plot indicates that production of these compounds is more dominant in certain accessions, or perhaps even individual plants within each accession.

The final cluster consists solely of two replicates of SR5, and is the most extreme within the sample set. As mentioned previously, SR5 is a promising cultivar that appears to be very different, in many respects, from other accessions of *E. sativa*, such as its higher GSL and ITC contents.

##### 3.3.3.2 ‘Day 3’

Fig. 3 illustrates the PCA for ‘Day 3’ samples. 46.23% of variation within the data is explained by the analysis, with correlations becoming significant at *r* = ±0.434 (*p* ⩽ 0.05). Variation between replicates on this sampling day is much reduced, with replicates for each respective accession being much closer together spatially than on ‘Day 0’. Clusters are much less well defined, but can be broadly characterized into three groups.

The first cluster (Fig. 3a) consists of just two replicates of SR12 which seem to be relative outliers overall (much like SR5 on ‘Day 0’). These replicates are generally characterized by the prevalence of a set of VOCs correlated in this same direction: 3-hexenal (**C15** *r* = 0.741), (E)-2-hexenal (**C8** *r* = 0.742), 3-octyne (**C17** *r* = 0.671), and hexyl ITC (**C29** *r* = 0.634). Some of these compounds are more indicative of ‘Day 0’ VOCs, and may indicate a predisposition in this accession for these types of compounds to be produced for a prolonged period of time after the initial production stimuli. This may have implications for the food and agricultural industries, as it implies that beneficial health compounds can be sustained during shelf life by appropriate selection of varieties. The impact of the industrial supply chain on phytochemical content has yet to be properly determined.

The second cluster is central to the plot in Fig. 3a, stretching towards the lower left. This loose cluster contains all three replicates of SR6, SR5 and SR19, with two replicates of SR14, and a single replicate of SR12. Genotypically, this cluster represents the most diverse range of accessions.

The final cluster to the right side of Fig. 3a includes all three replicates of SR2 and SR3, and one replicate of SR14. Compounds correlating in this direction include (E)-4-oxohex-2-enal (**C1** *r* = 0.571), 2-hexenal (**C7** *r* = 0.582), 1-penten-3-ol (**C3** *r* = 0.584), (E)-2-pentenal (**C10** *r* = 0.835), 1-penten-3-one (**C4** *r* = 0.650), vinylfuran (**C42** *r* = 0.714), 2-ethyl-furan (**C26** *r* = 0.830) and tetrahydrothiophene (**C38** *r* = 0.647). These compounds are indicative of the ‘Day 3’ profile highlighted in the previous section.

##### 3.3.3.3 ‘Day 7’

Fig. 4 displays the PCA plots for ‘Day 7’, where 58.69% of variation was explained by the data, and correlations became significant at *r* = ±0.434 (*p* ⩽ 0.05). In Fig. 4a, samples can be broadly divided into two clusters, left and right of the *y*-axis. Samples on the left are tightly clustered and those to the right are thinly spread along the *x*-axis. SR3, SR6 and SR5 have replicates contained in both clusters, which is unusual, considering that on ‘Day 3’ they were relatively close together. SR3 is the most extreme example, with replicates spread very far apart, indicating increased variability under controlled environmental conditions.

The dense left cluster contains all replicates of SR14, SR12 and SR19, two replicates of SR5 and one replicate of SR3, but it contained no significant correlations with any compounds.

The cluster on the right of the plot contains all replicates of SR2, two replicates of SR3 and SR6, and one replicate of SR5. Many of the compounds seem to be skewed on the scores plot in the direction of the SR3 and SR2 replicates. This suggests that these accessions have maintained a degree of VOC diversity later in the shelf-life period than the other accessions, which may be attributable to differing genetics. Compounds correlated with these samples along the first principal component include: 2-hexenal (**C7** *r* = 0.920), 3-hexenal (**C15** *r* = 0.862), (E)-4-oxohex-2-enal (**C1** *r* = 0.840), 1-penten-3-ol (**C3** *r* = 0.837), (E)-2-pentenal (**C10** *r* = 0.791), (E)-2-hexenal (**C8** *r* = 0.803), 3-octyne (**C17** *r* = 0.697), 2-ethyl-furan (**C26** *r* = 0.663). The presence of aldehyde compounds in this group is again indicative of extensive lipid breakdown.

### 3.4 Implications of detected VOCs for rocket quality and human nutrition

Between 1.0 ± 0.6% (SR3) and 6.8 ± 2.2% (SR5) of volatile compounds produced on ‘Day 0’ are isothiocyanates that may have potential benefits for human health (Verkerk et al., 2009). Although present at relatively low levels compared to other VOCs detected, isothiocyanates have been shown to be efficacious in eliciting health benefits at very low concentrations in previous *in vitro* studies with other crops (10 μM; Hanlon, Webber, & Barnes, 2007). Abundance of these compounds declined with storage to less than one percent on ‘Day 7’, indicating that, by the time the rocket leaves reach the consumer, it is possible that there has been a substantial drop in nutritional value. Processes that precede packaging, such as harvest, transport and washing, can be especially harsh on leaves, although some forms of stress can induce secondary metabolite production (Mewis et al., 2012). Thus, combined effects of storage and handling during the supply chain should be examined in relation to VOC loss.

## 4 Conclusions

Our results represent rocket plants grown under controlled environment conditions, and may differ from plants that are grown under variable field conditions. Many pre-harvest factors may affect the abundance and ratios of VOCs, such as disease status, soil type, light intensity and water status (Varming et al., 2004). Our method was established in order to minimize these stress effects and produce results based on genotypic and post-harvest storage factors, rather than pre-harvest environmental variation. Changes in phytochemical content between different growing environments are poorly documented in rocket species, and non-existent for VOCs. Future work will examine the effects that these factors may have on the crop and the end consumer, by conducting experiments in a commercial, field trial setting.

Our study has shown that there are significant differences amongst cultivars in the relative abundance of volatiles that they produce post-harvest and their retention during storage. Therefore, there is scope for plant breeders to consider basing selections, at least in part, on post-harvest VOC profiles in order to select for improved flavour and nutritionally valuable traits. By assessing such data in the supply chain, alongside phytochemical screening at harvest, rocket lines can feasibly be bred to limit losses of important VOCs, such as ITCs. More research is needed to fully understand where exactly these losses occur within the supply chain, and what (if anything) can be done to mitigate such potential losses.

The sampling methodology established here might also have potential applications within the food processing industry as part of quality assurance methods for rocket leaves. Sampling itself is straightforward and requires little specialist knowledge. Samples could be taken at critical points during processing to assess effects on VOC profiles with the aim of keeping ITC losses to a minimum. Most, if not all, producers routinely take and store samples of different batches of rocket salad to assess visual traits. In the near future, it is likely that quality assurance will expand beyond this to both phytochemical and volatile traits of many crops, not just rocket salad.

## Funding

Luke Bell is supported by a BBSRC Case Award (Reference BB/J012629/1) in partnership with Elsoms Seeds Ltd. (Spalding, United Kingdom) and Bakkavor Group Ltd. (Spalding, United Kingdom).

Work conducted at the Cardiff University is supported by the European Union Seventh Framework Program for research, technological development and demonstration, as part of the QUAFETY (Quality/Safety) collaborative project (KBBE 2011 2.4-01).

## Conflict of interest

The authors declare no conflict of interest.

## Figures and Tables

**Supplementary Fig. S1:**
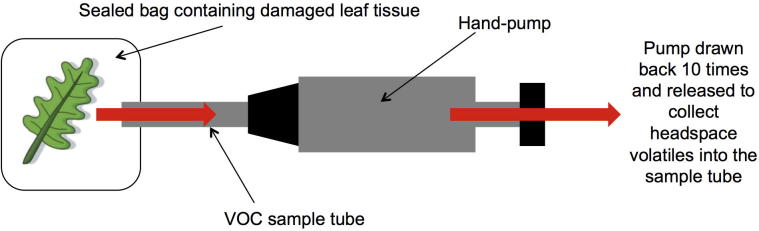


**Table 1:** VOC compounds putatively identified in rocket accessions by TD-GC–MS-TOF with retention indices, CAS registry number, chemical group, and references for identifications in previous studies.

Compound number	Compound name	RI	CAS No.	Chemical group	References
C1	(E)-4-oxohex-2-enal⁎	989	EPA-374042	Aldehyde	Sanda, Zacek, Streinz, Dracinsky, and Koutek (2012)
C2	4-Isothiocyanato-1-butene⁎	1021	3386-97-8	Isothiocyanate	Guo, Yang, Wang, Guo, and Gu (2014)
C3	1-Penten-3-ol⁎	677	616-25-1	Alcohol	Fernandes et al. (2009)
C4	1-Penten-3-one⁎	683	1629-58-9	Ketone	Servili, Selvaggini, Taticchi, Esposto, and Montedoro (2003)
C5	2-(1,1-Dimethylethyl)-1H-indole⁎	1582	1805-65-8	Aromatic compound	–
C6	2-Methyl-2-butenal⁎	702	1115-11-3	Aldehyde	Starkenmann (2003)
C7	2-Hexenal⁎	865	505-57-7	Aldehyde	Servili et al. (2003)
C8	(E)-2-hexenal	858	6728-26-3	Aldehyde	Blazevic and Mastelic (2008)
C9	(Z)-2-penten-1-ol⁎	782	1576-95-0	Alcohol	Rohloff and Bones (2005), Servili et al. (2003)
C10	(E)-2-pentenal⁎	760	1576-87-0	Aldehyde	Fernandes et al. (2009)
C11	(E,E)-2,4-hexadienal⁎	933	142-83-6	Aldehyde	Farag, Ryu, Sumner, and Paré (2006)
C12	5-Ethyl-2(5H)-furanone⁎	1003	2407-43-4	Furanone	Buttery and Takeoka (2004)
C13	3-Ethyl-1,5-octadiene⁎	961	EPA-114877	Alkene	Rohloff and Bones (2005)
C14	3-Hexen-1-ol	872	3681-71-8	Alcohol	Ruther and Kleier (2005), Blazevic and Mastelic (2008, 2009)
C15	3-Hexenal⁎	810	4440-65-7	Aldehyde	Tandon, Baldwin, and Shewfelt (2000)
C16	(Z)-3-hexenal⁎	898	6789-80-6	Aldehyde	Buttery and Takeoka (2004)
C17	3-Octyne⁎	921	15232-76-5	Alkyne	–
C18	3-Pentanone⁎	691	96-22-0	Ketone	Servili et al. (2003)
C19	5-Methyl-4-hexen-3-one⁎	1073	13905-10-7	Ketone	–
C20	4-Methylpentyl isothiocyanate	1184	17608-07-0	Isothiocyanate	Beevi, Mangamoori, Subathra, and Edula (2010)
C21	5-Nonanone oxime⁎	791	14475-42-4	Imine	–
C22	O-methyloxime-butanal⁎	672	31376-98-4	Imine	–
C23	1-Isothiocyanato-3-methyl-butane⁎	1082	628-03-5	Isothiocyanate	Zhao et al. (2007)
C24	Ethylidene-cyclopropane⁎	564	18631-83-9	Cycloalkane	Bajpai, Rahman, and Kang (2008)
C25	Dimethyl-sulphide⁎	570	75-18-3	Sulphur compound	Manchali, Chidambara Murthy, and Patil (2012)
C26	2-Ethyl-furan⁎	696	3208-16-0	Aromatic compound	Farag et al. (2006)
C27	3-Methyl-furan⁎	605	930-27-8	Aromatic compound	–
C28	3-Methyl-hexadecane⁎	1658	6418-43-5	Alkane	Wang, Xing, Chin, Ho, and Martin (2001)
C29	*n*-Hexyl-isothiocyanate⁎	1223	4404-45-9	Isothiocyanate	Fernandes et al. (2009)
C30	2-Oxo-hexanoic acid methyl ester⁎	1085	6395-83-1	Ester	–
C31	*n*-Pentyl isothiocyanate⁎	1120	629-12-9	Isothiocyanate	Grob and Matile (1980)
C32	Oxalic acid diallyl ester⁎	808	EPA-309229	Ester	–
C33	Iberverin⁎	1355	505-79-3	Isothiocyanate	Munday and Munday (2004)
C34	Propanoic acid anhydride⁎	1086	123-62-6	Acid anhydride	–
C35	4-Methyl-2-(2-methyl-propenyl)-pyridine⁎	1424	104188-16-1	Pyridine derivative	–
C36	Pyrrolidine-1-dithiocarboxylic acid 2-oxocyclopentyl ester⁎	1391	147723-50-0	Sulphur aromatic compound	–
C37	3-Ethyl-thiophene⁎	885	1795-01-3	Sulphur aromatic compound	–
C38	Tetrahydrothiophene	826	110-01-0	Sulphur aromatic compound	Blazevic and Mastelic (2008)
C39	[Unknown 2]	693	-	–	–
C40	[Unknown 8]	1129	-	–	–
C41	[Unknown 9]	991	-	–	–
C42	Vinylfuran⁎	726	1487-18-9	Aromatic compound	–

⁎ Previously unreported in *Eruca sativa*.

**Table 2:** Means of percentage relative abundance with standard error and significant differences (*p* ⩽ 0.05) of volatile compounds detected in the headspace of rocket accessions. Values expressed as percentage of the total volatiles detected per sample.

VOC	Cultivar and day of sampling (% of total volatiles detected)																				
SR2			SR3			SR5			SR6			SR12			SR14			SR19		
0	3	7	0	3	7	0	3	7	0	3	7	0	3	7	0	3	7	0	3	7
C1	22.4 ± 0.4^abc^	30.5 ± 2.7^bc^	25.6 ± 6.2^abc^	14.8 ± 2.9^ab^	49.9 ± 0.9^c^	22.5 ± 4.9^abc^	10.5 ± 8.6^ab^	nd^a^	1.9 ± 1.0^ab^	4.7 ± 3.8^ab^	20.6 ± 2.9^ab^	17.9 ± 7.3^ab^	2.5 ± 2.0^ab^	19.7 ± 4.1^ab^	3.2 ± 1.5^ab^	13.4 ± 8.6^ab^	19.8 ± 4.9^ab^	9.3 ± 4.1^ab^	12.3 ± 5.1^ab^	8.4 ± 1.4^ab^	3.3 ± 1.9^ab^
C2	nd^a^	nd^a^	nd^a^	nd^a^	nd^a^	nd^a^	<0.1 ± <0.1^b^	nd^a^	nd^a^	0.1 ± <0.1^c^	nd^a^	nd^a^	nd^a^	nd^a^	nd^a^	nd^a^	nd^a^	nd^a^	nd^a^	nd^a^	nd^a^
C3	1.2 ± 0.1^ab^	1.5 ± 0.2^b^	1.3 ± 0.2^ab^	1.5 ± 0.3^b^	0.7 ± 0.3^ab^	1.4 ± 0.7^ab^	0.5 ± 0.1^ab^	nd^a^	nd^a^	1.2 ± 0.2^ab^	0.7 ± 0.1^ab^	0.5 ± 0.2^ab^	1.5 ± 0.2^b^	nd^a^	nd^a^	1.3 ± 0.1^ab^	nd^a^	nd^a^	1.2 ± 0.2^ab^	nd^a^	nd^a^
C4	1.9 ± 0.1^a^	4.7 ± 1.0^a^	4.2 ± 1.0^a^	2.0 ± 0.2^a^	2.6 ± 0.9^a^	4.3 ± 2.2^a^	1.3 ± 0.5^a^	1.0 ± 0.6^a^	0.9 ± 0.6^a^	1.4 ± 0.3^a^	1.8 ± 0.3^a^	1.8 ± 0.3^a^	1.8 ± 0.2^a^	1.2 ± 0.2^a^	0.6 ± 0.2^a^	1.9 ± 0.2^a^	3.0 ± 0.4^a^	2.7 ± 1.5^a^	2.2 ± 0.3^a^	1.8 ± 0.7^a^	0.5 ± 0.1^a^
C5	nd^a^	nd^a^	nd^a^	nd^a^	nd^a^	nd^a^	nd^a^	nd^a^	nd^a^	<0.1 ± <0.1^a^	nd^a^	nd^a^	<0.1 ± <0.1^a^	nd^a^	nd^a^	nd^a^	nd^a^	nd^a^	<0.1 ± <0.1^a^	nd^a^	nd^a^
C6	nd^a^	nd^a^	nd^a^	<0.1 ± <0.1^a^	nd^a^	nd^a^	nd^a^	nd^a^	nd^a^	<0.1 ± <0.1^a^	nd^a^	nd^a^	0.1 ± <0.1^a^	nd^a^	nd^a^	<0.1 ± <0.1^a^	nd^a^	nd^a^	<0.1 ± <0.1^a^	nd^a^	nd^a^
C7	4.3 ± 0.5^abc^	6.5 ± 1.6^abc^	4.8 ± 0.5^abc^	7.7 ± 2.1^abc^	5.8 ± 0.5^abc^	4.9 ± 2.2^abc^	4.7 ± 1.6^abc^	1.4 ± 1.1^ab^	1.2 ± 0.9^ab^	7.3 ± 1.8^abc^	2.5 ± 0.6^abc^	2.1 ± 0.8^abc^	9.9 ± 1.7^c^	2.4 ± 0.7^abc^	0.4 ± 0.2^a^	7.7 ± 2.4^abc^	2.2 ± 0.8^abc^	nd^a^	8.9 ± 0.9^bc^	1.0 ± 0.5^ab^	0.3 ± 0.2^a^
C8	0.7 ± 0.2^ab^	nd^a^	0.1 ± 0.1^a^	3.1 ± 1.6^ab^	nd^a^	0.6 ± 0.2^ab^	0.4 ± 0.1^a^	nd^a^	nd^a^	3.5 ± 1.5^ab^	0.2 ± 0.1^a^	0.1 ± <0.1^a^	5.5 ± 1.7^b^	0.4 ± 0.2^a^	nd^a^	3.4 ± 1.9^ab^	nd^a^	nd^a^	1.9 ± 1.0^ab^	nd^a^	nd^a^
C9	0.1 ± <0.1^ab^	nd^a^	nd^a^	0.2 ± 0.1^bc^	nd^a^	nd^a^	nd^a^	nd^a^	nd^a^	0.2 ± 0.1^bc^	nd^a^	nd^a^	0.3 ± 0.1^c^	nd^a^	nd^a^	0.2 ± 0.1^bc^	nd^a^	nd^a^	0.2 ± <0.1^bc^	nd^a^	nd^a^
C10	1.0 ± <0.1^ab^	3.0 ± 0.6^bc^	2.8 ± 0.7^bc^	1.2 ± 0.2^abc^	4.0 ± 0.8^c^	2.1 ± 0.8^abc^	1.0 ± 0.3^ab^	0.8 ± 0.6^ab^	0.6 ± 0.5^ab^	1.0 ± 0.1^ab^	1.1 ± 0.1^ab^	1.1 ± 0.3^ab^	1.3 ± 0.2^abc^	0.9 ± 0.1^ab^	0.4 ± 0.2^ab^	1.3 ± 0.1^abc^	1.5 ± 0.7^abc^	nd^a^	1.5 ± 0.1^abc^	1.2 ± 0.4^abc^	0.4 ± 0.1^ab^
C11	0.4 ± 0.1^ab^	nd^a^	nd^a^	1.0 ± 0.4^ab^	nd^a^	0.2 ± 0.2^ab^	0.2 ± 0.1^ab^	nd^a^	nd^a^	1.4 ± 0.6^ab^	nd^a^	nd^a^	1.6 ± 0.5^b^	0.3 ± 0.1^ab^	nd^a^	1.1 ± 0.5^ab^	nd^a^	nd^a^	0.8 ± 0.2^ab^	nd^a^	nd^a^
C12	0.2 ± <0.1^ab^	nd^a^	nd^a^	0.2 ± 0.1^ab^	nd^a^	0.1 ± <0.1^a^	<0.1 ± <0.1^a^	nd^a^	nd^a^	0.4 ± <0.1^bc^	nd^a^	nd^a^	0.4 ± <0.1^bc^	nd^a^	nd^a^	0.5 ± 0.1^c^	nd^a^	nd^a^	0.4 ± 0.1^bc^	nd^a^	nd^a^
C13	nd^a^	nd^a^	nd^a^	nd^a^	nd^a^	nd^a^	nd^a^	nd^a^	nd^a^	nd^a^	0.4 ± <0.1^ab^	0.2 ± 0.1^ab^	<0.1 ± <0.1^a^	0.9 ± 0.4^b^	nd^a^	nd^a^	0.4 ± 0.2^ab^	0.2 ± 0.2^ab^	nd^a^	0.1 ± 0.1^a^	nd^a^
C14	39.3 ± 5.4^a^	1.0 ± 2.9^a^	25.0 ± 2.5^a^	36.9 ± 14.1^a^	12.5 ± 4.0^a^	12.5 ± 3.8^a^	14.1 ± 1.9^a^	9.9 ± 5.0^a^	20.7 ± 14.0^a^	17.0 ± 1.4^a^	23.9 ± 7.8^a^	17.0 ± 6.6^a^	14.3 ± 1.1^a^	10.4 ± 3.6^a^	4.7 ± 2.1^a^	15.7 ± 1.9^a^	38.0 ± 5.5^a^	8.6 ± 4.3^a^	24.2 ± 5.2^a^	17.5 ± 3.4^a^	4.9 ± 3.2^a^
C15	12.2 ± 2.6^abc^	4.8 ± 2.8^abc^	6.8 ± 2.8^abc^	18.7 ± 5.6^c^	3.4 ± 1.6^abc^	12.9 ± 5.4^abc^	6.9 ± 1.6^abc^	2.1 ± 1.4^ab^	1.7 ± 1.2^ab^	15.3 ± 1.8^abc^	6.1 ± 1.2^abc^	5.2 ± 2.0^abc^	16.5 ± 1.2^bc^	7.3 ± 2.5^abc^	2.1 ± 0.5^ab^	11.7 ± 3.3^abc^	nd^a^	nd^a^	17.4 ± 3.2^bc^	2.0 ± 1.4^ab^	nd^a^
C16	nd^a^	nd^a^	nd^a^	nd^a^	nd^a^	nd^a^	nd^a^	nd^a^	nd^a^	0.1 ± <0.1^a^	nd^a^	nd^a^	1.4 ± 1.1^a^	nd^a^	nd^a^	nd^a^	nd^a^	nd^a^	nd^a^	nd^a^	nd^a^
C17	0.1 ± <0.1^a^	nd^a^	nd^a^	<0.1 ± <0.1^a^	nd^a^	<0.1 ± <0.1^a^	<0.1 ± <0.1^a^	nd^a^	nd^a^	<0.1 ± <0.1^a^	nd^a^	nd^a^	<0.1 ± <0.1^a^	0.1 ± 0.1^a^	nd^a^	nd^a^	nd^a^	nd^a^	<0.1 ± <0.1^a^	nd^a^	nd^a^
C18	0.2 ± <0.1^a^	nd^a^	0.1 ± <0.1^a^	0.1 ± 0.1^a^	nd^a^	0.1 ± 0.1^a^	0.1 ± <0.1^a^	0.2 ± 0.1^a^	0.1 ± <0.1^a^	0.1 ± <0.1^a^	0.2 ± 0.1^a^	nd^a^	0.1 ± <0.1^a^	0.1 ± <0.1^a^	nd^a^	0.2 ± 0.1^a^	0.2 ± 0.1^a^	nd^a^	0.3 ± 0.1^a^	nd^a^	nd^a^
C19	nd^a^	nd^a^	nd^a^	nd^a^	nd^a^	nd^a^	0.9 ± 0.4^a^	1.2 ± 0.9^a^	nd^a^	2.3 ± 1.4^a^	nd^a^	nd^a^	3.3 ± 1.4^a^	nd^a^	<0.1 ± <0.1^a^	1.8 ± 1.4^a^	nd^a^	nd^a^	2.2 ± 1.8^a^	nd^a^	nd^a^
C20	1.2 ± 0.2^a^	1.5 ± 0.6^a^	1.5 ± 0.6^a^	0.7 ± 0.3^a^	nd^a^	0.7 ± 0.2^a^	4.3 ± 1.0^a^	3.4 ± 1.6^a^	0.3 ± 0.1^a^	1.1 ± 0.3^a^	1.6 ± 0.5^a^	0.6 ± 0.2^a^	1.3 ± 0.2^a^	1.3 ± 0.4^a^	0.1 ± <0.1^a^	0.8 ± 0.1^a^	nd^a^	nd^a^	2.4 ± 0.5^a^	4.7 ± 2.4^a^	0.3 ± 0.2^a^
C21	0.1 ± <0.1^a^	14.3 ± 4.7^abcd^	11.7 ± 3.5^a^	0.1 ± <0.1^a^	8.7 ± 2.8^abc^	12.9 ± 9.9^abcd^	0.2 ± <0.1^a^	36.9 ± 7.5^cd^	27.2 ± 5.7^abcd^	0.1 ± <0.1^a^	11.1 ± 2.8^abcd^	24.7 ± 10.0^abcd^	<0.1 ± <0.1^a^	15.8 ± 2.5^abcd^	36.8 ± 2.6^cd^	0.1 ± <0.1^a^	13.3 ± 1.2^abcd^	23.5 ± 7.1^abcd^	0.3 ± <0.1^ab^	30.4 ± 4.8^bcd^	40.9 ± 3.5^d^
C22	nd^a^	7.6 ± 3.4^abc^	8.2 ± 4.1^abc^	nd^a^	4.2 ± 1.8^ab^	17.7 ± 9.9^abc^	0.2 ± 0.1^a^	27.1 ± 7.6^abc^	43.3 ± 12.8^abc^	nd^a^	9.9 ± 3.3^abc^	21.3 ± 10.8^abc^	nd^a^	25.9 ± 6.4^abc^	48.2 ± 3.0^c^	nd^a^	6.3 ± 3.4^abc^	46.0 ± 19.4^bc^	nd^a^	30.9 ± 0.6^abc^	48.9 ± 2.6^c^
C23	nd^a^	nd^a^	nd^a^	nd^a^	nd^a^	nd^a^	<0.1 ± <0.1^ab^	nd^a^	nd^a^	<0.1 ± <0.1^ab^	nd^a^	nd^a^	<0.1 ± <0.1^ab^	nd^a^	nd^a^	nd^a^	nd^a^	nd^a^	<0.1 ± <0.1^b^	nd^a^	nd^a^
C24	<0.1 ± <0.1^ab^	nd^a^	nd^a^	<0.1 ± <0.1^a^	nd^a^	nd^a^	<0.1 ± <0.1^a^	nd^a^	nd^a^	0.1 ± <0.1^ab^	nd^a^	nd^a^	0.1 ± <0.1^ab^	nd^a^	nd^a^	0.1 ± <0.1^b^	nd^a^	nd^a^	<0.1 ± <0.1^ab^	nd^a^	nd^a^
C25	nd^a^	1.1 ± 0.7^ab^	1.5 ± 0.8^ab^	nd^a^	nd^a^	nd^a^	nd^a^	1.8 ± 0.6^ab^	0.3 ± 0.2^a^	nd^a^	0.4 ± 0.2^a^	1.5 ± 0.8^ab^	nd^a^	2.6 ± 0.8^ab^	2.1 ± 1.3^ab^	nd^a^	10.0 ± 5.2^b^	7.8 ± 2.7^ab^	nd^a^	nd^a^	nd^a^
C26	1.9 ± 0.3^abc^	5.0 ± 0.6^c^	3.3 ± 0.7^abc^	1.3 ± 0.3^abc^	4.6 ± 1.4^bc^	2.1 ± 0.9^abc^	2.8 ± 0.4^abc^	1.8 ± 0.8^abc^	0.7 ± 0.4^ab^	3.2 ± 0.3^abc^	2.0 ± 0.3^abc^	1.5 ± 0.4^abc^	1.9 ± 0.3^abc^	1.4 ± 0.3^abc^	0.6 ± 0.3^ab^	2.7 ± 0.4^abc^	2.7 ± 0.7^abc^	1.9 ± 1.0^abc^	2.1 ± 0.1^abc^	1.4 ± 0.5^abc^	0.4 ± <0.1^a^
C27	<0.1 ± <0.1^c^	nd^a^	nd^a^	<0.1 ± <0.1^b^	nd^a^	nd^a^	nd^a^	nd^a^	nd^a^	<0.1 ± <0.1^b^	nd^a^	nd^a^	<0.1 ± <0.1^b^	nd^a^	nd^a^	<0.1 ± <0.1^b^	nd^a^	nd^a^	nd^a^	nd^a^	nd^a^
C28	nd^a^	nd^a^	1.3 ± 0.6^b^	nd^a^	nd^a^	nd^a^	nd^a^	nd^a^	0.1 ± <0.1^a^	nd^a^	nd^a^	nd^a^	nd^a^	nd^a^	nd^a^	nd^a^	nd^a^	nd^a^	nd^a^	nd^a^	nd^a^
C29	0.4 ± 0.1^a^	nd^a^	nd^a^	0.2 ± 0.1^a^	nd^a^	0.1 ± 0.1^a^	1.8 ± 0.7^b^	nd^a^	nd^a^	0.4 ± <0.1^a^	0.3 ± 0.1^a^	nd^a^	0.3 ± <0.1^a^	0.4 ± 0.2^a^	nd^a^	0.2 ± 0.1^a^	nd^a^	nd^a^	0.2 ± 0.1^a^	nd^a^	nd^a^
C30	10.4 ± 1.4^cd^	nd^a^	nd^a^	7.0 ± 2.9^abc^	nd^a^	4.7 ± 2.1^abc^	6.2 ± 1.1^abc^	nd^a^	nd^a^	10.8 ± 1.3^cd^	3.8 ± 0.4^abc^	1.0 ± 0.4^ab^	12.1 ± 1.6^cd^	3.7 ± 1.6^abc^	0.6 ± 0.1^ab^	16.7 ± 3.5^d^	nd^a^	nd^a^	8.9 ± 1.8^bcd^	nd^a^	nd^a^
C31	nd^a^	nd^a^	nd^a^	0.1 ± <0.1^a^	nd^a^	nd^a^	0.4 ± 0.2^b^	nd^a^	nd^a^	0.1 ± <0.1^a^	nd^a^	nd^a^	0.1 ± <0.1^ab^	nd^a^	nd^a^	0.1 ± <0.1^ab^	nd^a^	nd^a^	0.1 ± <0.1^a^	nd^a^	nd^a^
C32	nd^a^	nd^a^	nd^a^	nd^a^	nd^a^	nd^a^	nd^a^	nd^a^	nd^a^	nd^a^	nd^a^	nd^a^	3.3 ± 1.4^b^	nd^a^	nd^a^	nd^a^	nd^a^	nd^a^	nd^a^	nd^a^	nd^a^
C33	nd^a^	nd^a^	nd^a^	<0.1 ± <0.1^a^	nd^a^	nd^a^	0.1 ± 0.1^b^	nd^a^	nd^a^	<0.1 ± <0.1^a^	nd^a^	nd^a^	<0.1 ± <0.1^a^	nd^a^	nd^a^	<0.1 ± <0.1^a^	nd^a^	nd^a^	<0.1 ± <0.1^a^	nd^a^	nd^a^
C34	nd^a^	nd^a^	nd^a^	nd^a^	nd^a^	nd^a^	nd^a^	nd^a^	nd^a^	2.2 ± 0.9^ab^	nd^a^	nd^a^	3.9 ± 0.5^b^	nd^a^	nd^a^	3.1 ± 1.5^b^	nd^a^	nd^a^	nd^a^	nd^a^	nd^a^
C35	nd^a^	nd^a^	nd^a^	0.1 ± <0.1^a^	nd^a^	nd^a^	nd^a^	nd^a^	nd^a^	0.2 ± <0.1^ab^	nd^a^	nd^a^	0.5 ± 0.1^b^	nd^a^	nd^a^	0.2 ± 0.1^ab^	nd^a^	nd^a^	0.2 ± 0.1^ab^	nd^a^	nd^a^
C36	1.5 ± 0.9^a^	7.6 ± 3.3^a^	nd^a^	2.4 ± 1.1^a^	nd^a^	nd^a^	41.7 ± 16.8^b^	11.2 ± 2.9^a^	0.4 ± 0.2^a^	15.8 ± 7.0^ab^	12.1 ± 6.2^a^	2.9 ± 1.3^a^	7.4 ± 3.2^a^	4.8 ± 3.3^a^	nd^a^	8.2 ± 2.5^a^	nd^a^	nd^a^	11.2 ± 1.6^a^	nd^a^	nd^a^
C37	nd^a^	nd^a^	nd^a^	nd^a^	nd^a^	nd^a^	nd^a^	nd^a^	nd^a^	<0.1 ± <0.1^c^	nd^a^	nd^a^	<0.1 ± <0.1^b^	nd^a^	nd^a^	nd^a^	nd^a^	nd^a^	nd^a^	nd^a^	nd^a^
C38	0.3 ± <0.1^a^	1.6 ± 0.4^ab^	1.4 ± 0.2^ab^	0.2 ± 0.1^a^	3.0 ± 1.2^b^	0.1 ± 0.1^a^	1.2 ± 0.1^ab^	1.2 ± 0.3^ab^	0.5 ± 0.2^ab^	0.4 ± 0.1^ab^	0.9 ± 0.4^ab^	0.5 ± 0.2^ab^	0.2 ± 0.1^a^	0.4 ± 0.3^ab^	nd^a^	0.3 ± <0.1^a^	2.3 ± 1.1^ab^	nd^a^	0.5 ± <0.1^ab^	0.5 ± 0.3^ab^	0.1 ± 0.1^a^
C39	nd^a^	nd^a^	nd^a^	0.1 ± <0.1^a^	nd^a^	nd^a^	nd^a^	nd^a^	nd^a^	0.1 ± <0.1^a^	nd^a^	nd^a^	0.1 ± <0.1^a^	nd^a^	nd^a^	<0.1 ± <0.1^a^	nd^a^	nd^a^	<0.1 ± <0.1^a^	nd^a^	nd^a^
C40	0.1 ± <0.1^a^	nd^a^	nd^a^	0.3 ± 0.1^a^	nd^a^	nd^a^	0.3 ± 0.1^a^	0.1 ± <0.1^a^	nd^a^	0.3 ± <0.1^a^	0.5 ± 0.2^a^	nd^a^	0.3 ± 0.1^a^	nd^a^	nd^a^	0.2 ± 0.1^a^	nd^a^	nd^a^	0.3 ± 0.1^a^	nd^a^	nd^a^
C41	nd^a^	nd^a^	nd^a^	nd^a^	nd^a^	nd^a^	nd^a^	nd^a^	nd^a^	9.0 ± 0.2^b^	nd^a^	nd^a^	7.7 ± 0.4^b^	nd^a^	nd^a^	6.9 ± 3.0^b^	nd^a^	nd^a^	nd^a^	nd^a^	nd^a^
C42	<0.1 ± <0.1^a^	0.3 ± 0.1^ab^	0.3 ± 0.1^ab^	<0.1 ± <0.1^a^	0.7 ± 0.3^b^	0.1 ± 0.1^a^	<0.1 ± <0.1^a^	nd^a^	nd^a^	<0.1 ± <0.1^a^	<0.1 ± <0.1^a^	<0.1 ± <0.1^a^	<0.1 ± <0.1^a^	<0.1 ± <0.1^a^	<0.1 ± <0.1^a^	0.1 ± <0.1^a^	0.2 ± 0.1^ab^	nd^a^	0.1 ± <0.1^a^	<0.1 ± <0.1^a^	<0.1 ± <0.1^a^

nd = not detected.

Superscript letters in the same row indicate differing levels of significance for each respective cultivar across all sampling days (ANOVA Tukey’s HSD test; (*p* = ⩽0.05)).
